# Assessment of knowledge, attitudes, and practices of bushmeat value chain actors in Nigeria toward mpox and other zoonoses

**DOI:** 10.3389/fvets.2025.1556573

**Published:** 2025-05-07

**Authors:** Otto Vianney Muhinda, Adeyinka Jeremy Adedeji, Abraham Albert Zirra, Laibané Dieudonné Dahourou, Ayodele O. Majekodunmi, Yakubu Joel Atuman, Toyin Olubade, Mathew Sabah, Benjamin Emikpe

**Affiliations:** ^1^Food and Agriculture Organisation of the United Nations, Abuja, Nigeria; ^2^National Veterinary Research Institute (NVRI), Vom, Nigeria; ^3^Institut des Sciences de l’Environnement et du Développement Rural (ISEDR), Université Daniel Ouezzin Coulibaly, Souri, Burkina Faso; ^4^Department of Veterinary and Pest Control Services, Federal Ministry of Agriculture and Food Security (FMAFS), Abuja, Nigeria; ^5^School of Veterinary Medicine, Kwame Nkrumah University of Science and Technology, Kumasi, Ghana

**Keywords:** bushmeat trade, zoonotic diseases, mpox, Nigeria, knowledge attitudes practices

## Abstract

**Background:**

The bushmeat trade is a vital source of protein and income in Nigeria but presents significant public health risks due to its potential zoonotic disease transmission. Despite these risks, there has been limited exploration of the knowledge, attitudes, and practices (KAP) of individuals involved in the bushmeat value chain in Nigeria, particularly concerning food safety and transmission of wildlife related zoonoses such as mpox and others.

**Methods:**

A cross-sectional survey was conducted across three Nigerian states - Lagos, Rivers, and Bauchi - selected for their significant bushmeat trade activities and burden of zoonoses such as mpox and Lassa Fever. Using purposive sampling, data were collected from 238 participants (74 hunters, 115 traders, and 49 consumers) through structured questionnaires using Kobotools. Knowledge, attitudes, and practices of these value chain actors relating to disease transmission risk, handling and consumption of wildlife were evaluated.

**Results:**

Knowledge of zoonotic disease transmission was limited, particularly among hunters (8.1%), traders (10.4%), and consumers (12.2%). Attitudes toward zoonotic disease risk were largely risky, with most actors underestimating the health risks associated with bushmeat handling. Results of practices of the value chain actors, revealed poor hand hygiene practices and poor usage of personal protective equipment (PPE) such as after wildlife handling, further amplified the public health risks. Educational level emerged as a significant predictor of knowledge and attitudes, but not practices, with tertiary-educated participants displaying significantly higher knowledge and positive attitudes (*p* < 0.05).

**Conclusion:**

This study reveals critical knowledge deficits and risky attitudes and practices within the bushmeat value chain. Results indicate urgent need for targeted, culturally sensitive, public health interventions to improve knowledge of mpox and other zoonoses identification and transmission, attitudes toward the risk of wildlife as reservoirs of mpox mpox and other zoonoses. And finally, improvement of hygienic practices and usage of appropriate PPE along the bushmeat value chain.

## Introduction

Bushmeat, defined as the meat of wild or non-domesticated animals harvested for food, is a vital source of protein and income in many developing countries (1). The global bushmeat trade sits at the intersection of food security, cultural practices, economic necessity, and public health concerns. As human populations encroach on wildlife habitats and demand for bushmeat persists, the risk of zoonotic disease transmission from these sources increases. Bushmeat markets have been identified as hotspots for emerging and re-emerging pathogens, posing significant public health threats (2, 3, 33–35).

The World Health Organization (WHO) has declared several public health emergencies linked to zoonoses, including the Ebola and COVID-19, both which were associated with bushmeat handling and markets (4–6). The more recent mpox (formerly called monkey pox) public health emergency further highlighted the risks associated with bushmeat trade. Mpox was reported in Nigeria in 2017 after nearly four decades of absence since the last report in 1978 (7). Mpox is currently endemic in Nigeria reported in 35 out 36 States of the country with over 1,240 confirmed human cases and 9 deaths from 2017 to date (NCDC Mpox SITREP Epi 44, Nov 3 2024). In addition, Nigerians have been reported to be responsible for travel associated spread of mpox to some countries in Asia, Europe and America (8).

Nigeria is one of the largest consumers of bushmeat in West Africa, with an extensive and diverse bushmeat trade estimated at 100 metric tonnes annually (9). This high demand leads to indiscriminate and unregulated hunting practices, increased human-wildlife interaction, and the risk of zoonotic disease transmission (10). A study in Nigeria reported that despite the public and worldwide anxiety due to the COVID-19 pandemic, bushmeat markets trade activities continued to thrive without government intervention or restrictions (11). While previous studies have explored the ecological impacts of bushmeat trade (1, 9), there is a notable lack of research on the knowledge, attitudes, and practices (KAP) of value chain actors regarding zoonotic disease transmission along the bushmeat value chain.

Studies in Nigeria have highlighted significant gaps in the knowledge, attitudes, and practices (KAP) regarding bushmeat at individual steps within the value chain (hunters or traders or consumers) (12, 13), however, few studies have considered the entire value chain as one entity, to understand linkages and variations which may affect risk of disease transmission at each step. This study therefore aims to address this knowledge gap by examining the KAP of these stakeholders regarding risk of zoonoses transmission across the value chain and mpox specifically.

## Method

### Study approach and design

This study employed a cross-sectional survey design to analyze KAP concerning mpox and zoonoses among bushmeat value chain actors. A cross-sectional approach, ideal for capturing data at a specific point in time, is widely used in health behavior research to understand variations across demographic and occupational groups (14). This approach allowed the study to efficiently capture actor specific data on zoonotic disease awareness, perceived risks, and behavioral practices.

### Study area

**Figure 1:**
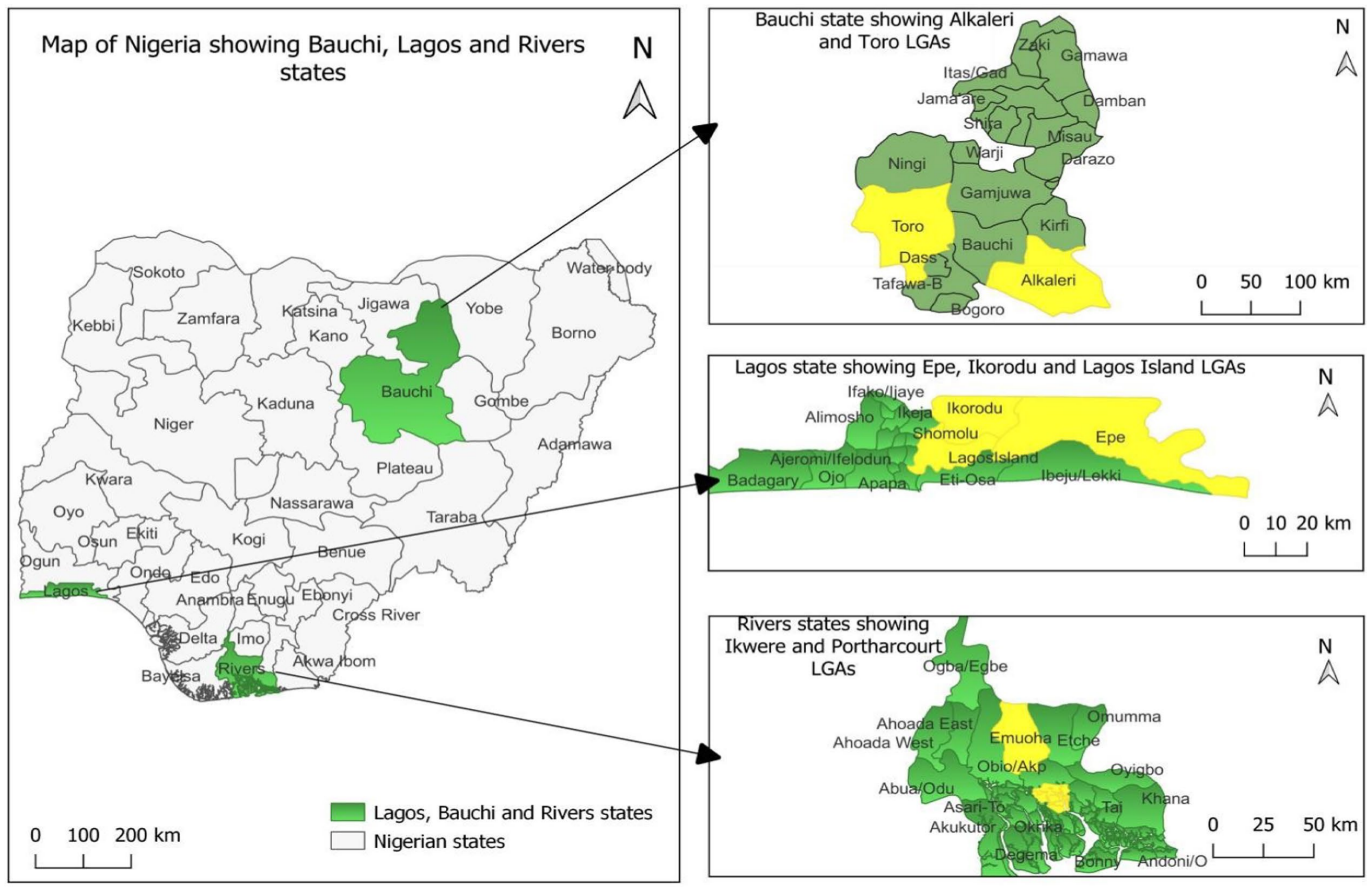
Map of Nigeria showing selected states and local government areas of the study.

This study was conducted in three administrative states of Nigeria, namely Lagos, Rivers, and Bauchi States (Figure 1). The three states were purposefully selected based on the burden of confirmed mpox human cases, evidence of bushmeat trade activities, and the presence of wildlife game reserves. Data from Nigeria Centre for Disease Control and Prevention (NCDC), shows Lagos State had 240 human mpox cases the highest number of confirmed human cases in Nigeria at Epi week 15, 16th April 2023 (NCDC Mpox SITREP 2023). Lagos is also the hub of illegal bushmeat trade in Nigeria because of presence of multiple portal entries (11, 15, 16). Lagos State has a population of 12,550,598 people (2016) and literacy level of 94.1%.^1^

Rivers has an estimated population of 7,303,924 people as at 2016 and adult literacy is 80.3% according to Nigerian Bureau Statistics (see Footnote 1) Rivers State has the second highest number of confirmed human mpox cases in the country with 92 confirmed cases as at Epi week 15, 16th April 2023 (NCDC Mpox SITREP 2023). Interestingly, *Orthopoxvirus* antibodies have been detected in rodent populations in some communities with confirmed human mpox cases in Rivers State (11). Furthermore, the Rumuodara Forest game reserve is also located in the State.

Bauchi State located in North Eastern Nigeria with a population of 6,537,314 (2016) and adult literacy level of 39.4%. Bauchi State has also reported confirmed human cases of mpox and the state is a high burden State for Lassa fever virus. Three game reserves are located in Bauchi State, i.e., Yankari, Sumu, and Lame-Burra game reserves (17).

Previously, studies have reported bushmeat market activities in these selected states of Nigeria (16, 18, 19). The three States also represent different ecological regions of Nigeria. Lagos is in the tropical rainforest region, River State is located in the mangrove ecological region, and Bauchi State is in the Savanna ecological zone of the country. Hunters’ hubs were also selected for this study namely Alaba-Rago in Ojo LGA and Ndele in Emohua LGA of Rivers States.

### Sampling strategy

The sampling strategy integrated purposive and random sampling approaches. Purposive sampling was employed to select Lagos, Rivers, and Bauchi states based on their high burden of mpox cases, active bushmeat markets, and varied ecological zones. This approach a was grounded in the principles of purposive sampling, where locations directly impacting the research variables are strategically selected. Random sampling within these states ensured unbiased selection of participants from bushmeat markets and hunters’ hub, contributing to generalizable findings regarding the KAP of bushmeat value chain actors across regions.

### Sample size

The study was conducted across 12 Local Government Areas (LGAs) in three selected states in Nigeria. A total of 29 bushmeat market visits were carried out involving 12 bushmeat markets or sale points, with minimum of 2 visits per market over a one month, and one hunters’ hub was visited per state. A total of 238 participants consented and were selected, consisting of 115 traders, 74 hunters, and 49 consumers from three aforementioned States: Lagos, Rivers, and Bauchi. Each actor group was purposively sampled to represent the diversity of roles within the bushmeat trade, as previous studies have shown that KAP can significantly vary based on the position within the value chain (2). This sample size was deemed sufficient to allow meaningful analysis of distinct perspectives, which is vital in understanding how knowledge levels and practices differ among hunters, traders, and consumers. As diverse stakeholder groups in the bushmeat trade may have unique motivations and daily practices’, capturing this range was essential for a comprehensive KAP assessment.

### Data collection

A structured questionnaire, the primary data collection instrument, was carefully designed to capture both quantitative and qualitative data. The questionnaire included sections on socio-demographic information, knowledge of mpox and other zoonoses, attitudes toward mpox and other zoonoses, and bushmeat handling practices. Each section was crafted to assess different aspects of KAP, offering a holistic view of zoonotic disease awareness and risk behavior in the bushmeat value chain. For instance, the knowledge section asked participants questions on zoonotic disease awareness and mpox symptoms, while the attitudes section examined their perceptions of zoonoses such as mpox risk and willingness to adjust behavior if disease risks were known. The practices section focused on daily bushmeat handling and hygiene routines, crucial for understanding zoonotic transmission potential. The questionnaire was administered through the Kobocollect app, an Android-based open-source tool known for enhancing data accuracy and efficiency in field studies by reducing manual entry errors (20). This digital format also allowed for real-time data collection and minimized delays in the data processing workflow.

### Data collection procedure

Data collection took place over four weeks, allowing sufficient time to conduct in-depth interviews with bushmeat value chain actors at bushmeat markets and hunters’ hubs. A team of trained interviewers conducted the surveys, ensuring cultural sensitivity and accuracy, as they were fluent in local languages and dialects. Participants were recruited through multiple market visits where each market was visited three times to capture a range of participants and reduce sampling bias. Interviews were conducted onsite, with verbal informed consent obtained prior to participation.

### Grading and scoring system

To evaluate knowledge, attitudes, and practices (KAP) among bushmeat value chain actors, a structured scoring system was implemented. Knowledge was assessed through questions about zoonotic diseases, with each correct answer scoring one point; cumulative scores were categorized as good (7–10 points), moderate (4–6 points), or poor (0–3 points). Attitudes were measured on a Likert scale, with positive responses scoring +1, neutral responses scoring 0, and negative responses scoring-1, resulting in positive (7–10), neutral (4–6), or negative (0–3) classifications. Practices were evaluated based on selfreported adherence to safe handling and hygiene procedures, with each correct practice scoring +1.

Total practice scores were categorized as good (7–10 points), moderate (4–6 points), or poor (0–3 points). This grading provided a consistent method to assess knowledge, attitudes, and practices among actors in the bushmeat trade as used by Deku et al. (21).

### Data analysis

Data collected using the Kobocollect app was downloaded, transferred into a Microsoft Excel spreadsheet version 19 for data cleaning. The cleaned data was analyzed using R Software. After data cleaning and preparation, descriptive statistics were used to summarize demographic characteristics and KAP variables for bushmeat value chain actors.

Chi-square tests were used to examine the associations between categorical variables, specifically exploring the relationship between roles (hunters, traders, and consumers) and KAP outcomes. Multinomial logistic regression was conducted to determine predictors of knowledge levels, using factors such as education, age, and role within the value chain. Additionally, binary logistic regression assessed predictors of attitudes toward zoonotic disease prevention. These statistical methods provided a robust framework for understanding the influence of demographic and occupational factors on KAP variables. All inferential statistics were performed at 5% significance level or 95% confidence interval.

### Ethical consideration

The study received ethical clearance from the National Veterinary Research Institute Animal Ethics Committee (NVRI/AEC/03/133/23), ensuring compliance with established ethical standards for research involving human participants. Permissions were also obtained from the respondents and respective Ministries of Agriculture in each surveyed state, ensuring adherence to regional policies.

## Results

### Socio-demographic characteristics

The socio-demographic characteristics of bushmeat hunters, traders, and consumers are presented in Table 1. The majority of hunters (60.8%) and traders (59.1%) were between 30–44 years old, while consumers were more evenly distributed across age groups, with 44.9% being 45 years and above. Gender distribution varied significantly among the groups, with all hunters (100%) being male, while traders had a more balanced distribution (43.5% male, 56.5% female). Consumers were predominantly male (71.4%). Marital status was similar across groups, with most participants being married (hunters: 77.0%, traders: 79.1%, consumers: 87.75%).

**Table 1:** Socio-demographics information of bushmeat hunters, traders, and consumers.

Variable	Categories	Hunter (*n* = 74)	Trader (*n* = 115)	Consumer (*n* = 49)
Age of respondents	18–29	11 (14.9%)	12 (10.4)	7 (14.3%)
	30–44	45 (60.8%)	68 (59.1)	20 (40.8%)
	45 and above	18 (24.3%)	35 (30.4)	22 (44.9%)
Gender of respondents	Male	74 (100%)	50 (43.5)	35 (71.4%)
	Female	0 (0%)	65 (56.5)	14 (28.6%)
Marital status	Married	57 (77.0%)	91 (79.1)	43 (87.75%)
	Single	15 (20.3%)	15 (13.0)	6 (12.24%)
	Widowed	1 (1.4%)	4 (3.5)	0 (0%)
	Divorced	1 (1.4%)	4 (3.5)	0 (0%)
Educational level	None	14 (18.9%)	4 (3.5)	8 (16.32%)
	Primary School	19 (25.7%)	26 (22.6)	11 (22.44%)
	Secondary School	40 (54.1%)	72 (62.6)	16 (32.65%)
	University Degree	1 (1.4%)	9 (7.8)	9 (18.36%)
	Postgraduate Studies	0 (0%)	3 (2.6)	5 (10.20%)
Primary occupation	Business	29 (39.2%)	11 (9.6)	18 (36.7%)
	Civil Servant	18 (24.3%)	56 (48.7)	9 (18.36%)
	Farming	12 (16.2%)	36 (31.3)	5 (10.20%)
	Health Practitioner	14 (18.9%)	12 (10.4)	2 (4.08%)
	Others	0 (0%)	0 (0%)	12 (24.48%)
Years involved with bushmeat trade	1–4	29 (39.2%)	11 (9.6)	4 (8.16%)
	5–14	18 (24.3%)	56 (48.7)	12 (24.48%)
	15–29	12 (16.2%)	36 (31.3)	12 (24.48%)
	30 and above	14 (18.9%)	12 (10.4)	21 (42.85%)
Location of respondents	Bauchi	30 (40.5%)	33 (28.7)	13 (26.5%)
	Lagos	14 (18.9%)	41 (35.7)	2 (4.1%)
	Rivers	29 (39.2%)	39 (33.9)	34 (69.4%)

Educational levels varied, with secondary education being the most common among hunters (54.1%) and traders (62.6%), while consumers had a more diverse educational background, including higher percentages of university (18.36%) and postgraduate (10.20%) degrees. Primary occupations differed among groups. The most common occupation for hunters was business (39.2%), for traders it was civil service (48.7%), and for consumers it was business (36.7%). Years involved in the bushmeat trade varied, with hunters mostly having 1–4 years of experience (39.2%), traders 5–14 years (48.7%), and consumers 30 years and above (42.85%). The study participants were from three locations: Bauchi, Lagos, and Rivers. Hunters were primarily from Bauchi (40.5%) and Rivers (39.2%), traders were more evenly distributed across the three locations, and consumers were predominantly from Rivers (69.4%) (Table 1).

### Purpose of engagement in bushmeat trade

The data presented in Table 2 elucidates the various motivations and customer demographics associated with the engagement of hunters and traders in the bushmeat trade. The motivations for hunter and trader involvement in the bushmeat trade were primarily economic, with profitability cited by 64.9% of hunters and 86.1% of traders. Inheritance also played a significant role, involving 50.0% of hunters and 32.2% of traders, while lack of employment influenced 47.3% of hunters and 19.1% of traders. Minimal percentages attributed their engagement to cultural or religious reasons (2.7% of hunters, 0.9% of traders) (Table 2).

**Table 2:** Purpose of hunters and traders’ engagement in bushmeat trade.

Variable	Categories	Hunters (*n* = 74)	Traders (*n* = 115)
Reason for your involvement in bushmeat trade	Lack of employment	35 (47.3%)	22 (19.1%)
	Cultural and religious reasons	2 (2.7%)	1 (0.9%)
	Inherited	37 (50.0%)	37 (32.2%)
	Profitable	48 (64.9%)	99 (86.1%)
What are the reasons why your customers buy bushmeat?	Food	70 (94.6%)	114 (99.3%)
	Medicine	37 (50.0%)	43 (37.4%)
	Pet	3 (4.0%)	6 (5.2%)
	Security/charms	2 (2.7%)	1 (0.9%)
	Artifacts	0 (0%)	0 (0%)
Who are your customers?	Consumers	52 (70.3%)	100 (87%)
	Caterers	36 (48.6%)	61 (53.0%)
	Traders/Wholesalers/Retailers	61 (82.4%)	74 (64.3%)
	Civil Servants/Government Officials	4 (5.4%)	26 (22.6%)
	Traditional Medicine Seller	23 (31.1%)	32 (27.8%)

Customer demand was predominantly for food, reported by 94.6% of hunters’ customers and 99.3% of traders’ customers, while 50.0 and 37.4% of customers, respectively, purchased bushmeat for medicinal purposes. Minor reasons included use as pets (live wildlife) (4.0% hunters, 5.2% traders) and for security/charms (2.7% hunters, 0.9% traders) (Table 2).

Customer profiles revealed diverse demographics, with both hunters and traders serving direct consumers (70.3% hunters, 87.0% traders), caterers (48.6% hunters, 53.0% traders), and traditional medicine sellers (31.1% hunters, 27.8% traders). These results underscored bushmeat’s multifaceted role in food security, traditional medicine, and economic survival across varied customer bases (Table 2).

### Knowledge of bushmeat value chain actors concerning mpox and other zoonoses

The knowledge levels of bushmeat value chain actors regarding mpox and other zoonoses varied considerably across hunters, traders, and customers (Table 3). When asked if wildlife could be carriers of zoonotic diseases, only 22.97% of hunters and 11.30% of traders recognized this potential, compared to 53.06% of customers. A significant proportion of respondents were unsure, with 54.05% of hunters, 62.61% of traders, and 30.61% of customers indicating they did not know (Table 3).

**Table 3:** Knowledge of bushmeat value chain actors concerning mpox and other zoonoses.

Variables	Responses	Hunter (*n* = 74)	Traders (*n* = 115)	Customer (*n* = 49)
Knowledge of wildlife being carriers of zoonotic diseases	Yes, they are carriers of disease	17 (22.97)	13 (11.30)	26 (53.06)
	No, they are not carriers of disease	17 (22.97)	29 (25.22)	8 (16.33)
	I do not know whether they are carriers of disease or not	40 (54.05)	72 (62.61)	15 (30.61)
	mpox	9 (12.16)	13 (11.30)	20 (40.82)
Diseases wildlife/bushmeat transmit to humans	Rabies	6 (8.11)	2 (1.74)	14 (28.57)
	Ebola/Marburg COVID-19	11 (14.86)	7	18 (36.73)
		6 (8.1)	2 (1.74)	8 (16.33)
	Influenza	3 (4.05)	2 (1.74)	8 (16.33)
	Lassa fever	0 (0%)	3 (2.60)	20 (40.82)
	Salmonella	5 (6.76)	0 (0%)	6 (12.2)
	Anthrax	4 (5,41)	1 (0.90)	6 (12.2)
	Malaria	4 (5.41)	0 (0%)	1 (2.04)
	Tuberculosis	8 (10.81)	3 (2.60)	11 (22.45)
Heard of mpox disease	Yes	45 (60.81)	94 (81.74)	34 (69.39)
	No	29 (39.19)	21 (18.26)	15 (30.61)
Mode of contracting mpox disease	Eating monkey meat contact with infected persons.	2 (2.70)	9 (7.8)	8 (16.33)
		7 (9.46)	18 (15.66) 3 (2.61)	3 (6.12)
	From infected animals	2 (2.70)	20 (17.39)	2 (4.08)
	I do not know	32 (43.24)	9 (7.8)	23 (46.94)
	sexual intercourse	0 (0%)	1 (0.87)	0 (0%)
Knowledge of symptoms of mpox	Yes	23 (31.1)	35 (30.43)	21 (42.9)
	No	25 (33.8)	46 (40.00)	9 (18.4)
	I do not know	25 (35.1)	34 (29.56)	19 (38.8)
Symptoms of mpox	Boil-like rash	10 (13.51)	7 (6.09)	6 (12.2)
	Chicken-pox like rash	4 (5.41)	2 (1.74)	3 (6.12)
	Pox lesions	4 (5.41)	2 (2.61)	10 (20.41)
	Rashes	11 (14.86)	17 (14.78)	0 (0%)
	Fever	2 (2.7)	2 (1.74)	3 (6.12)
	Cough	0 (0%)	0 (0%)	1 (2.04)
	Headache	0 (0%)	0 (0%)	1 (2.04)
	Nausea	0 (0%)	0 (0%)	1 (2.04)
	Do not know	3 (4.05)	0 (0%)	3 (6.12)

Regarding specific zoonotic diseases transmitted by wildlife, awareness of mpox was highest among customers (40.82%), followed by hunters (12.16%) and traders (11.30%). Similarly, customer knowledge of diseases like Ebola/Marburg (36.73%) and rabies (28.57%) was notably higher than that of hunters and traders. Awareness of other diseases, such as Lassa Fever, was also significantly higher among customers (40.82%) than among hunters or traders, where it was rarely mentioned (Table 3).

Awareness of mpox was widespread, with 60.81% of hunters, 81.74% of traders, and 69.39% of customers having heard of it. However, knowledge of mpox transmission modes was limited. Only 9.46% of hunters and 15.66% of traders identified contact with infected persons as a possible mode, while a notable 43.24% of hunters and 17.39% of traders admitted uncertainty (Table 3). Interestingly, almost all hunters, hunters and customers claimed sexual activities are not associated with mpox transmission. Recognition of mpox symptoms were relatively low, with only 31.1% of hunters, 30.43% of traders, and 42.9% of customers indicating familiarity. Among symptoms, “boil-like rash” was most recognized by hunters (13.51%), while “pox lesions” were identified by 20.41% of customers but only by 5.41% of hunters and 2.61% of traders (Table 3).

### Source of knowledge of mpox disease

**Figure 2:**
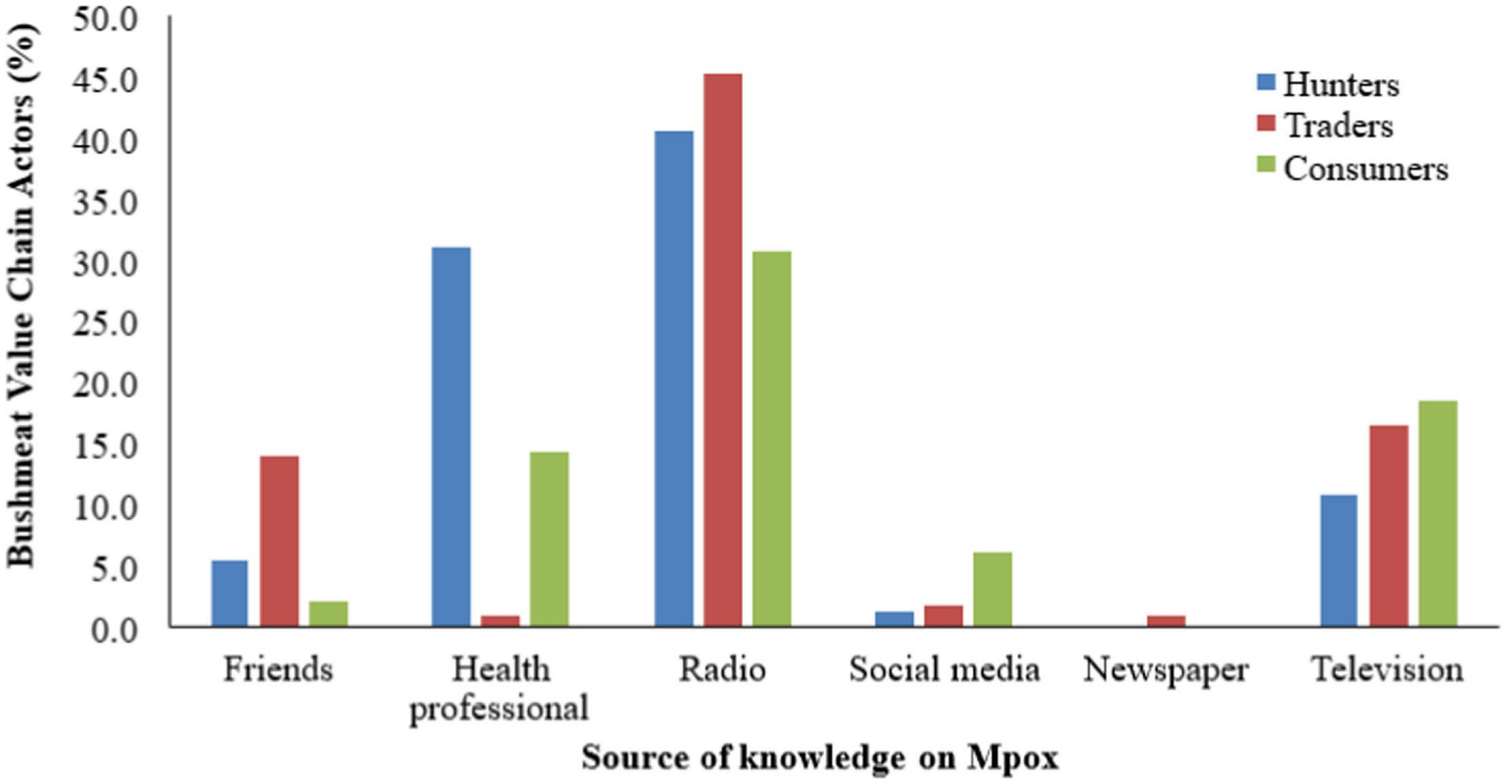
Response to source of bushmeat value chain actors’ knowledge of mpox disease.

The result displayed in Figure 2 depicts the sources of knowledge about mpox among bushmeat value chain actors. Radio was the most common source, cited by 45.2% of traders, 40.5% of hunters, and 34.7% of consumers. Health professionals were a significant source for hunters (32.4%) and consumers (20.4%), but less so for traders (2.6%). While television was source of information for 26.1% of traders, 24.5% of consumers, and 17.6% of hunters. Friends were cited by 12.2% of traders, 5.4% of hunters, and 4.1% of consumers. Social media was noted by 10.2% of consumers but was minimal for hunters (2.7%) and traders (0.9%). Newspapers were the least used, with no significant usage reported across the groups (Figure 2).

### Attitude of bushmeat value chain actors toward mpox and other zoonoses

The attitudes of bushmeat value chain actors toward the health risks associated with mpox and other zoonotic diseases showed significant variations in risk perception and behavior (Table 4). A majority of hunters (66.2%) and customers (55.1%) believed there was no risk from eating or handling wildlife, while 48.7% of traders held the same view. Notably, 43.5% of traders considered the risk to be medium, compared to just 6.8% of hunters and 10.2% of customers. Few respondents recognized a high risk, with 8.11% of hunters, 1.7% of traders, and 10.2% of customers identifying it as such (Table 4).

**Table 4:** Attitude of bushmeat value chain actors concerning mpox and other zoonoses.

Variables	Responses	Hunter (*n* = 74)	Traders (*n* = 115)	Customer (*n* = 49)
Presence of any health risks from eating or handling wildlife	No risk	49 (66.2)	56 (48.7)	27 (55.1)
	Little risk	14 (18.9)	7 (6.1)	12 (24.5)
	Medium risk	5 (6.8)	50 (43.5)	5 (10.2)
	High risk	6 (8.11)	2 (1.7)	5 (10.2)
Would stop eating wildlife if I knew there was risk of disease transmission	Yes	8 (16.32)	17 (14.00)	8 (16.32)
	No	41 (55.40)	35 (30.4)	41 (83.67)
	Do not know	0 (0%)	7 (6.1)	0 (0%)
Mpox is a disease of serious health concern to your colleagues involved in bushmeat trade	Yes	33 (44.6)	15 (13)	12 (24.5)
	No	33 (44.6)	65 (56.5)	21 (42.9)
	Do not know	7 (9.5)	35 (30.4)	16 (32.7)
Selling wildlife or their body parts is a health risk to this market	No risk	41 (55.4)	56 (48.7)	55.1
	Little risk	20 (27)	7 (6.1)	10.2
	Medium risk	7 (9.5)	50 (43.5)	24.5
	High risk	5 (6.8)	2 (1.7)	10.2

When asked if they would stop consuming wildlife if they knew of disease transmission risks, only 16.32% of hunters and customers stated they would, while the majority of customers (83.67%) and 55.4% of hunters indicated they would not. Traders were more divided, with 30.4% responding negatively.

Regarding whether mpox was considered a serious health concern, 44.6% of hunters viewed it as a significant issue, whereas 56.5% of traders disagreed. Among customers, 42.9% did not consider mpox a serious concern, with 32.7% expressing uncertainty. In terms of selling wildlife or their body parts as a health risk to the market, 55.4% of hunters, 48.7% of traders, and 55.1% of customers perceived no risk. However, a significant proportion of traders (43.5%) viewed the risk as medium, compared to 9.5% of hunters and 24.5% of customers. High-risk perception remained minimal across all groups.

### Report of zoonoses outbreak and its impact bushmeat trade activities

The responses of bushmeat value chain actors regarding the impact of report of zoonoses on the bushmeat trade reveal varied effects. The declaration ofmpox as a public health emergency had minimal impact, with only 2.70% of hunters, 9.57% of traders, and 16.32% of customers reporting it affected their trade. The vast majority across all groups comprising 97.27% of hunters, 90.43% of traders, and 83.67% of customers stated it did not affect their trade (Table 5).

**Table 5:** Bushmeat value chain actors’ response to zoonoses outbreak affecting the bushmeat trade.

Variables	Responses	Hunter (*n* = 74)	Traders (*n* = 115)	Customer (*n* = 49)
Mpox declared as a public health emergency has affected the trade of bush meat or wildlife	Yes, it affected	2 (2.70)	11 (9.57)	8 (16.32)
	No, it did not affect	72 (97.27)	104 (90.43)	41 (83.67)
Diseases that have affected my business bushmeat trade the most	Covid-19	39 (52.70)	52 (45.61)1	15 (30.61)
	Ebola	24 (32.43)	60 (52.63)	26 (53.06)
	Mpox	0 (0%)	0 (0%)	2 (4.08)
	None	11 (14.86)	2 (1.75)	0 (0%)

In terms of the diseases that had the greatest impact on their bushmeat businesses, COVID-19 was identified by 52.70% of hunters, 45.61% of traders, and 30.61% of customers as the most disruptive.

Ebola also significantly affected bushmeat trade, particularly for traders (52.63%) and customers (53.06%), while 32.43% of hunters reported it as a major issue. Mpox, by contrast, had little impact, with no hunters or traders reporting it as a significant factor and only 4.08% of customers citing it. A small percentage of hunters (14.86%) and traders (1.75%) stated that no disease had affected their business (Table 5).

### Practices of bushmeat value chain actors toward handling and hygiene

The practices of bushmeat value chain actors in handling wildlife varied across hunters, traders, and customers. All hunters (100%) reported involvement in wildlife hunting, compared to 52.2% of traders and only 6.1% of customers. A vast majority of customers (93.9%) and almost half of traders (47.8%) indicated no involvement in hunting (Table 6).

**Table 6:** Practices of bushmeat value chain actors in handling wildlife.

Variables	Responses	Hunter (*n* = 74)	Traders (*n* = 115)	Customer (*n* = 49)
Involved in wildlife hunting	Yes	74 (100%)	60 (52.2)	2 (6.1)
	No	0 (0%)	55 (47.8)	46 (93.9)
Do you use protective clothing when handling/slaughtering butchering wildlife	Yes	14 (18.93)	26 (22.6)	3 (6.1)
If yes, what type of protective clothing?	No	59 (79.73)	87 (75.7)	46 (93.88)
Gloves		5 (6.76)	14 (12.1)	2 (4.08)
Face mask		2 (2,70)	4 (3.48)	1 (2.04)
Apron		5 (6.76)	12 (10.43)	3 (6.12)
Boot		3 (4.05)	5 (4.35)	2 (4.08)
Other (Additional clothing)		1 (1.35)	7 (6.09)	1 (2.04)
Ever handled any bushmeat with boil/pox-like lesions	Do not Know	1 (1.4)	2 (1.74)	6 (12.24)
	Yes	4 (5.4)	1 (0.87)	0 (0%)
	No	63 (85.1)	112 (97.39)	43 (87.76)
Wildlife species handed with pox-like lesions	African pouched rat	2 (2.7)	0 (0%)	0 (0%)
	Fox	0 (0%)	0 (0%)	0 (0%)
	Birds	0 (0%)	0 (0%)	0 (0%)
	Rat	0 (0%)	1 (0.87)	0 (0%)
	Squirrel	1 (1.35)	1 (0.87)	0 (0%)
	Monkey	2 (2.70)	0 (0%)	0 (0%)
Mode of handwashing after handling wildlife	Do not wash my hands until the end of the day	18 (24.32)	7 (6.09)	2 (4.08)
	Rarely wash my hands	15 (20.27)	1 (0.87)	4 (8.16)
	Wash your hands with soap & water and use hand sanitizer	4 (5.41)	7 (6.09)	34 (69.39)
	Wash your hands with soap & water every time wildlife is handled	28 (37.84)	64 (55.65)	8 (16.33)
	Wash your hands with water only every time wildlife is handled	9 (12.16)	36 (31.30)	0 (0%)
Consumption of wildlife/bushmeat	No	1 (1.35)	1 (0.87)	0 (0%)
	Yes	73 (98.65)	114 (99.13)	49 (100)
Mode of processing wildlife/bushmeat or their body parts for consumption	Drying	9 (12.16)	7 (6.09)	6 (12.24)
	Lightly cooked	2 (2.70)	7 (6.09)	0 (0%)
	Well cooked	61 (82.43)	99 (86.09)	40 (81.63)
	Others (Using liquor)	1 (1.35)	6 (5.2)	3 (6.12)

Regarding the handling of bushmeat with boil or pox-like lesions, 5.4% of hunters and 0.87% of traders admitted to handling such bushmeat, whereas none of the customers had this experience. Most respondents denied encountering these lesions, with 85.1% of hunters, 97.39% of traders, and 87.76% of customers stating they had not (Table 6). Among the few wildlife species reported with pox-like lesions, African pouched rat and monkey were noted by hunters (2.7% each), and squirrel by both hunters (1.35%) and traders (0.87%) (Table 6).

Handwashing practices after handling wildlife varied. A significant portion of hunters (24.32%) did not wash their hands until the end of the day, compared to 6.09% of traders and 4.08% of customers. While 37.84% of hunters and 55.65% of traders reported washing their hands with soap and water after each handling, this practice was less common among customers (16.33%). Additionally, 69.39% of customers used soap, water, and hand sanitizer, compared to only 5.41% of hunters and 6.09% of traders (Table 6). Also, all most the value chain actor claimed poor usage of personal protective equipment as gloves, facemask, apron and boots while handling bushmeat. This is most notable in customers (93.88), and hunters (79.73) compared to traders (75.7) (Table 6).

Almost all respondents reported consuming wildlife, with 98.65% of hunters, 99.13% of traders, and 100% of customers confirming consumption. The majority preferred their bushmeat well-cooked, including 82.43% of hunters, 86.09% of traders, and 81.63% of customers. Other methods of preparation included drying (12.16% of hunters, 6.09% of traders, and 12.24% of customers) and lightly cooking, though this was less common (2.7% of hunters, 6.09% of traders, and none of the customers). A small percentage across all groups reported using liquor as part of their processing methods (Table 6).

### Association between knowledge, attitude, and practices and roles of bushmeat value chain actors

The chi-square analysis in Table 7 uncovers a shared landscape of knowledge, attitudes and practices among bushmeat value chain actors namely hunters, traders, and consumers. The results indicated significant disparities in knowledge levels, with only a small percentage demonstrating good knowledge (2.7% of hunters in Bauchi, 1.4% in Lagos, and 4.1% in Rivers; traders reported 2.6, 4.3, and 3.5% respectively; consumers showed 4.1 2.0, and 4.1%). These differences in knowledge levels were statistically significant among the groups at different locations (χ^2^ = 108.25, *p* = 0.027) (Table 7). In terms of attitudes, positive responses were noted among a minority of participants (4.1% of hunters in Bauchi, 2.7% in Lagos, and 5.4% in Rivers, traders reported similar percentages), with a significant variation in attitudes across locations (χ^2^ = 143.96, *p* = 0.001). Regarding practices, good practices were reported at low rates (4.1% among hunters and varying percentages among traders and consumers), with a significant difference (χ^2^ = 444.49, *p* = 0.0001) in practices among the groups studied (Table 7).

**Table 7:** Chi-square analysis of knowledge, attitude, and practices among bushmeat value chain actors.

Category	Level	Hunters (*n* = 74)			Traders (*n* = 115)			Consumers (*n* = 49)			χ^2^ value	*p*-value
		Bauchi	Lagos	Rivers	Bauchi	Lagos	Rivers	Bauchi	Lagos	Rivers		
Knowledge	Good	2 (2.7%)	1 (1.4%)	3 (4.1%)	3 (2.6%)	5 (4.3%)	4 (3.5%)	2 (4.1%)	1 (2.0%)	2 (4.1%)	108.25	0.027*
	Moderate	12 (16.2%)	11 (14.9%)	10 (13.5%)	12 (10.4%)	14 (12.2%)	11 (9.6%)	6 (12.2%)	4 (8.2%)	5 (10.2%)		
	Poor	20 (27.0%)	19 (25.7%)	14 (18.9%)	15 (13.0%)	18 (15.7%)	16 (13.9%)	7 (14.3%)	6 (12.2%)	5 (10.2%)		
Attitude	Positive	3 (4.1%)	2 (2.7%)	4 (5.4%)	5 (4.3%)	6 (5.2%)	5 (4.3%)	3 (6.1%)	2 (4.1%)	3 (6.1%)	143.96	0.001*
	Neutral	9 (12.2%)	8 (10.8%)	7 (9.5%)	11 (9.6%)	12 (10.4%)	10 (8.7%)	6 (12.2%)	5 (10.2%)	4 (8.2%)		
	Negative	18 (24.3%)	16 (21.6%)	12 (16.2%)	14 (12.2%)	14 (12.2%)	14 (12.2%)	7 (14.3%)	7 (14.3%)	5 (10.2%)		
Practices	Good	3 (4.1%)	4 (5.4%)	2 (2.7%)	4 (3.5%)	5 (4.3%)	4 (3.5%)	3 (6.1%)	1 (2.0%)	3 (6.1%)	444.49	0.000*
	Moderate	10 (13.5%)	11 (14.9%)	9 (12.2%)	12 (10.4%)	14 (12.2%)	12 (10.4%)	5 (10.2%)	4 (8.2%)	5 (10.2%)		
	Poor	17 (23.0%)	14 (18.9%)	13 (17.6%)	13 (11.3%)	14 (12.2%)	15 (13.0%)	8 (16.3%)	6 (12.2%)	6 (12.2%)		

### Predictors of knowledge and positive attitude toward mpox and other zoonoses

A multinomial logistic regression was conducted to predict the knowledge level of mpox and other zoonoses among bushmeat value chain actors (Table 8). The analysis revealed that education level was the most significant predictor of knowledge levels. Participants with tertiary education were significantly more likely to have moderate (OR = 2.87, 95% CI: 1.11–7.42, *p* = 0.029) or good (OR = 5.26, 95% CI: 1.24–22.31, *p* = 0.024) knowledge levels compared to those with no formal education. There was also a trend toward higher knowledge levels among those with secondary education, although this did not reach statistical significance (moderate knowledge: OR = 2.13, 95% CI: 0.98–4.63, *p* = 0.057; good knowledge: OR = 3.45, 95% CI: 0.88–13.52, *p* = 0.075). Actor type, gender, and age group did not significantly predict knowledge levels. Traders and consumers did not differ significantly from hunters in their likelihood of having moderate or good knowledge. Similarly, gender and age group were not associated with significant differences in knowledge levels.

**Table 8:** Multinomial logistic regression predicting knowledge level of mpox and other zoonoses.

Variable	Knowledge level	Odds ratio	95% CI	*p*-value
Actor type (ref: Hunter; *n* = 74)				
Trader (*n* = 115)	Moderate	1.28	0.69–2.37	0.431
	Good	1.32	0.46–3.78	0.605
Consumer (*n* = 49)	Moderate	1.24	0.59–2.61	0.573
	Good	1.58	0.48–5.21	0.452
Gender Female (*n* = 79) vs. Male (*n* = 159)	Moderate	0.98	0.53–1.81	0.945
	Good	0.95	0.37–2.45	0.918
Age group (ref: 18–30 years)				
31–45 years (*n* = 133)	Moderate	1.15	0.59–2.24	0.682
	Good	1.42	0.47–4.29	0.537
46–60 years (*n* = 75)	Moderate	0.89	0.42–1.89	0.761
	Good	1.18	0.34–4.09	0.795
> 60 years	Moderate	0.76	0.21–2.75	0.675
	Good	0.95	0.11–8.32	0.963
Education level (ref: no formal; *n* = 26)				
Primary (*n* = 56)	Moderate	1.56	0.72–3.38	0.259
	Good	2.18	0.54–8.81	0.275
Secondary (*n* = 128)	Moderate	2.13	0.98–4.63	0.057
	Good	3.45	0.88–13.52	0.075
Tertiary (*n* = 27)	Moderate	2.87	1.11–7.42	0.029
	Good	5.26	1.24–22.31	0.024

CI, Confidence Interval; ref, reference category. The reference category for the dependent variable (Knowledge Level) is “Poor”.

A binary logistic regression was conducted to identify predictors of positive attitudes toward Mpox and other zoonoses prevention among bushmeat value chain actors (Table 9). The analysis revealed that education level and knowledge level were significant predictors of positive attitudes. Participants with tertiary education were significantly more likely to have positive attitudes compared to those with no formal education (OR = 2.47, 95% CI: 1.06–5.75, *p* = 0.036).

**Table 9:** Binary logistic regression predicting positive attitudes toward mpox and other zoonoses prevention among bushmeat value chain actors.

Predictor	Odds ratio	95% CI	*p*-value
Actor type (ref: Hunter; *n* = 74)			
Trader (*n* = 115)	1.24	0.68–2.26	0.482
Consumer (*n* = 49)	1.56	0.79–3.08	0.201
Gender (ref: Male; *n* = 159)			
Female (*n* = 79)	0.92	0.51–1.66	0.781
Age group (ref: 18–30; *n* = 30)			
31–45 (*n* = 133)	1.18	0.62–2.24	0.614
46–60 (*n* = 75)	1.35	0.66–2.76	0.411
> 60	1.72	0.52–5.68	0.373
Education Level (ref: No formal; *n* = 26)			
Primary (*n* = 56)	1.43	0.68–3.01	0.345
Secondary (*n* = 128)	1.89	0.92–3.88	0.084
Tertiary (*n* = 27)	2.47	1.06–5.75	0.036*
Knowledge Level (ref: Poor; *n* = 120)			
Moderate (*n* = 85)	1.76	0.98–3.16	0.058
Good (*n* = 23)	2.31	1.12–4.77	0.023*

CI, Confidence Interval; ref, reference category. *p < 0.05.

Knowledge level also emerged as a significant predictor of positive attitudes. Participants with good knowledge were 2.31 times more likely to have positive attitudes compared to those with poor knowledge (OR = 2.31, 95% CI: I. 12–4.77, *p* = 0.023). There was also a trend toward more positive attitudes among those with moderate knowledge, although this did not reach statistical significance (OR = 1.76, 95% Cl: 0.98–3.16, *p* = 0.058). Actor type, gender, and age group did not significantly predict positive attitudes (11 > 0.05). Traders and consumers did not differ significantly from hunters in their likelihood of having positive attitudes. Similarly, gender and age group were not associated with significant differences in attitudes (*p* > 0.05).

Species of wildlife hunted and traded by different value chain actors based on the questionnaire survey.

Based on data collected from the questionnaire survey, value chain actors claimed to hunted or traded different species of wildlife or bushmeat. The bushmeat value chain actors claimed to have hunted, traded or consumers 30 species of wildlife or bushmeat. The wildlife includes rodents (Grasscutter, African pouched rat, porcupine), Carnivores (Lion, leopards, Civet, hyena) and Primates (baboons, monkeys). Other wildlife claimed to be hunted or traded by large mammals (elephants, hippopotamus, buffalo) and reptiles (snakes, tortoise and monitor lizard) (Table 10). The most common hunted and traded category wildlife are rodents according to the bushmeat value chain actors (Table 10).

**Table 10:** Species of wildlife hunted and traded by different value chain actors based on questionnaire survey in the study area.

	Species	Hunter (*n* = 74)	Trader (*n* = 115)	Customer (*n* = 49)
1	Grasscutter	42 (56.8)	84 (73)	36 (73.5)
2	Buffalo	48 (64.9)	59 (51.3)	1 (2.0)
3	African Pouched rat	55 (74.3)	53 (46.1)	21 (42.9)
4	Squirrel	51 (68.9)	65 (56.5)	23 (46.9)
5	Monkey	14 (18.9)	13 (11.3)	11 (22.4)
6	Baboons	35 (47.3)	32 (27.8)	1 (2.0)
7	Duiker	5 (6.8)	_0	12 (24.5)
8	Elephant	28 (37.8)	24 (20.9)	0
9	Fox	0	0	5 (10.2)
10	Crocodile	30 (40.5)	27 (23.5)	13 (26.5)
11	Hare	4 (5.4)	2 (1.7)	13 (26.5)
12	Hippopotamus	13 (17.6)	9 (7.8)	0
13	Honey badger	16 (21.6)	4 (3.5)	5 (10.2)
14	Hyena	19 (25.7)	14 (12.2)	1 (2.0)
15	Pangolin	17 (23.0)	7 (6.1)	2 (4.1)
16	Jackal	1 (51)	78 (67.8)	0
17	Antelope	7 (9.5)	1 (0.9)	33 (67.3)
18	Leopard	2 (2.7)	1 (0.9)	3 (6.1)
19	Lion	39 (52.7)	26 (22.6)	0
20	Civet	48 (64.9)	59 (51.3)	3 (6.1)
21	Python/snakes	47 (63.5)	57 (49.6)	14 (28.6)
22	Monitor lizard	17 (23)	3 (2.6)	12 (24.5)
23	Vulture	33 (44.6)	32 (27.8)	0
24	Warthog/bush pig	11 (14.9)	1 (0.9)	10 (20.4)
25	Eagle	20 (27)	6 (5.2)	1 (2.0)
26	Bat	33 (44.6)	31 (27)	3 (6.1)
27	Tortoise	47 (63.5)	60 (52.2)	8 (16.3)
28	Porcupine	0	1 (0.9)	23 (46.9)
29	Ostrich	9 (12.2)	4 (3.5)	2 (2.0)
30	Buck	16 (21.6)	19 (16.5)	1 (2.0)

## Discussion

This current study focused on a comprehensive analysis of the knowledge, attitudes, and practices (KAP) of bushmeat value chain actors regarding mpox (formerly called monkeypox) and other zoonoses in Nigeria, offering crucial insights into the complex dynamics of the bushmeat trade and its potential public health implications. The findings revealed a multifaceted landscape of economic motivations, varied knowledge levels, and diverse risk perceptions among hunters, traders, and consumers. The economic imperative of the bushmeat trade emerged as a dominant theme, with profitability cited as the primary motivation for engagement by both hunters (64.9%) and traders (86.1%). This underscores the critical role of bushmeat in local livelihoods and food security, a finding consistent with previous studies in sub-Saharan Africa (9, 22). The inheritance of the trade practice (50.0% for hunters, 32.2% for traders) further emphasizes its cultural embeddedness and intergenerational significance. These findings align with research by Friant et al. (2) in Nigeria, which highlighted the socio-economic importance of bushmeat and the challenges this poses for disease control efforts. The strong economic motivation and cultural significance of the bushmeat trade suggest that any interventions aimed at reducing zoonotic disease risks must consider alternative livelihood options and culturally sensitive approaches to behavior change. Simply banning or restricting the trade without addressing these underlying factors is likely to be ineffective and may drive the practice underground, potentially increasing health risks.

Knowledge levels regarding zoonoses and mpox varied significantly across the value chain. Notably, consumers demonstrated higher awareness of wildlife as potential disease carriers (53.06%) compared to hunters (22.97%) and traders (11.30%). This disparity suggests a potential information gap within the supply chain, which could have implications for disease prevention and control strategies. The limited recognition of specific zoonotic diseases, particularly among hunters and traders, is concerning and aligns with findings from other studies in West Africa (2, 37). The study revealed that radio was the most common source of information about mpox disease across all actor groups, followed by health professionals for hunters and consumers. This finding highlights the importance of leveraging diverse communication channels for public health messaging. Similar results were reported by Suu-Ire et al. (23) in Ghana, where radio was found to be a crucial medium for disseminating information about zoonotic diseases in rural communities. The knowledge disparities across the value chain and the importance of radio as an information source suggest that targeted educational interventions using appropriate media channels could be effective in improving awareness and understanding of zoonotic diseases. Tailoring messages for different actor groups and utilizing radio broadcasts could enhance the reach and impact of public health communications. Another interesting knowledge gap is the poor knowledge of sexual activities and transmission of mpox by all the value actors. Currently, there is increasing evidence globally that sexual activities is a major factor in the transmission of mpox (24, 25).

Risk perception and attitudes toward mpox and other zoonoses revealed a complex picture. Most value chain actors, especially hunters (66.2%) and consumers (55.1%), perceived no health risks from handling or consuming wildlife. This low-risk perception, coupled with the economic importance of the trade, presents a significant challenge for public health interventions. This similar to study carried out in Nigeria by Alhaji et al. (26), that also reported low risk perception among bushmeat value chain actors regarding COVID-19. The reluctance to cease wildlife consumption even with knowledge of disease risks (83.67% of consumers, 55.4% of hunters) further complicates efforts to mitigate zoonotic disease transmission. These findings are consistent with research by Bonwitt et al. (27) in Sierra Leone and Meseko et al. (11) in Nigeria, which found that economic necessity often outweighed perceived health risks in bushmeat-related activities. The low-risk perception and reluctance to change behaviors despite awareness of potential health risks highlight the need for more effective risk communication strategies. These strategies should not only focus on increasing knowledge but also on addressing the underlying factors that influence risk perception and behavior, such as economic necessities and cultural beliefs.

Practices in wildlife handling and hygiene varied across the value chain, with concerning trends observed. For instance, 24.32% of hunters reported not washing their hands until the end of the day after handling wildlife, a practice that could significantly increase the risk of zoonotic disease transmission. This finding highlights the need for targeted interventions to improve hygiene practices among bushmeat handlers. Similar concerns were raised by Leroy et al. (28) in their study of Ebola outbreaks in Central Africa, emphasizing the critical role of hygiene practices in disease prevention. The poor hygiene practices observed, particularly among hunters, underscore the urgent need for practical, context-specific interventions to improve wildlife handling and personal hygiene. These interventions should be designed in collaboration with local communities to ensure they are feasible and culturally acceptable.

The impact of disease outbreaks on the bushmeat trade was notably differentiated. While COVID-19 and Ebola were reported to have significant effects on business, the declaration of mpox as a public health emergency had minimal impact on trade activities. This differential response to various disease outbreaks suggests complex factors influencing risk perception and trade practices, which warrant further investigation. The limited impact of mpox on trade practices contrasts with findings from studies on Ebola’s impact on bushmeat consumption in West Africa (29), possibly due to differences in perceived severity or media coverage of the diseases. The varied impact of different disease outbreaks on the bushmeat trade suggests that public health responses need to be tailored to specific diseases and contexts. Understanding the factors that influence the perceived severity of different diseases could help in developing more effective public health messaging and interventions.

The findings from the chi-square analysis of knowledge, attitudes, and practices among bushmeat value chain actors in Bauchi, Lagos, and Rivers states reveal significant variations. The results indicate that only a small percentage of participants demonstrated good knowledge (ranging from 1.4 to 4.1%) and positive attitudes (2.7 to 5.4%), with practices reported at low rates (4.1% among hunters). These findings align with studies conducted in other regions, such as Kinshasa, DRC, where vendors faced challenges related to declining bushmeat availability and profitability due to regulatory measures and market dynamics (36). A notable difference in the findings is the specific socioeconomic and cultural contexts of each state. Bauchi, being predominantly rural, may experience lower levels of education and awareness regarding bushmeat conservation compared to Lagos, a more urbanized area with greater access to information and resources. This urban–rural divide contributes to variations in knowledge and attitudes toward bushmeat consumption. Additionally, Rivers State’s coastal geography and more access economic resources which may influence dietary preferences and access to alternative protein sources, affecting both the demand for bushmeat and local actors’ perceptions of its sustainability.

The implications of these variations are significant, suggesting a need for targeted educational interventions that consider local cultural practices and economic dependencies on bushmeat trade. Previous studies have highlighted how regulatory measures can negatively impact profitability along the bushmeat commodity chain, indicating that any interventions should balance conservation efforts with the livelihoods of local communities (1). Understanding local perceptions of hunting as a cultural heritage can inform strategies that integrate community values into conservation initiatives (38). Practical interventions could include community-based education programs emphasizing sustainable hunting practices and the ecological importance of wildlife conservation. Collaborative efforts involving local authorities, NGOs, and community leaders could foster a more sustainable approach to bushmeat trade by promoting alternative livelihoods and enhancing market regulation.

The regression analyses revealed that education level was a significant predictor of both knowledge and positive attitudes toward mpox and other zoonoses prevention. Participants with tertiary education were more likely to have good knowledge (OR = 5.26, *p* = 0.024) and positive attitudes (OR = 2.47, *p* = 0.036) compared to those with no formal education. This finding underscores the importance of education in shaping health-related knowledge and attitudes, consistent with broader public health literature (30). The strong association between education level and both knowledge and attitudes suggest that improving access to education, particularly at higher levels, could have significant long-term benefits for public health in the context of zoonotic disease prevention. Integrating information about zoonoses and wildlife handling into educational curricula could be an effective strategy for long-term risk reduction. Results show that the bushmeat value chain has hunts, trades, and consumes at least thirty species of wildlife, which is worrisome and a threat to public health and wildlife conservation in Nigeria. Moreover, species of wildlife, such as rodents and primates, which are widely hunted or traded, are probable reservoirs of mpox (Table 10) (31). In addition, rodents and primates are putative reservoirs of zoonotic pathogens in the ecology (32).

## Conclusion

This study highlights the knowledge, attitudes, and practices (KAP) among bushmeat value chain actors in Nigeria concerning mpox and other zoonoses, revealing critical gaps, particularly among hunters and traders. While consumers show some awareness, hunters and traders exhibit significant knowledge deficits and low-risk perceptions about bushmeat handling, posing public health risks. Economic reasons is a driver of bushmeat trade further complicating health concerns, as income needs often take priority. These findings point to an urgent need for targeted educational interventions to improve knowledge and risk awareness, particularly via accessible channels like radio. Public health campaigns should promote safe handling practices, and efforts to offer economic alternatives could reduce reliance on bushmeat trade, supporting community resilience against zoonotic diseases. This study underscores the importance of culturally sensitive, health-focused interventions within the bushmeat sector.

### Recommendations

Based on the findings of this study, targeted educational programs are recommended to improve knowledge and risk awareness about zoonotic diseases among hunters and traders in Nigeria’s bushmeat sector. These interventions should leverage widely accessible channels, such as radio, to effectively communicate the risks associated with bushmeat handling and consumption.

Traders should receive practical hygiene training that emphasizes safe handling methods and the importance of personal hygiene to reduce disease transmission risks, alongside initiatives that provide alternative livelihood options to lessen economic dependencies on bushmeat. For consumers, awareness campaigns must focus on the health risks associated with bushmeat consumption, leveraging community-based programs that highlight safe food preparation practices while respecting cultural traditions.

## Data Availability

The original contributions presented in the study are included in the article/supplementary material, further inquiries can be directed to the corresponding author.

## Footnotes

^1^ https://nigeria.opendataforafrica.org/
